# Association between Life’s Essential 8 and cataract among US adults

**DOI:** 10.1038/s41598-024-63973-1

**Published:** 2024-06-07

**Authors:** Yang Meng, Zongbiao Tan, Abdulla Sawut, Lu Li, Changzheng Chen

**Affiliations:** 1 Department of Ophthalmology, Renmin Hospital of Wuhan University, 238 Jiefang Road, Wuhan, China; 2 Department of Gastroenterology, Renmin Hospital of Wuhan University, 238 Jiefang Road, Wuhan, China

**Keywords:** Life's Essential 8, Cardiovascular health, Cataract, Lifestyle, NHANES, Medical research, Outcomes research

## Abstract

Currently, a comprehensive assessment of the relationship between ideal cardiovascular health (CVH) indicators and cataract risk is lacking. Life’s Essential 8 (LE8) is the latest concept proposed by the American Heart Association to comprehensively reflect CVH status. LE8 includes four health behaviors (diet, physical activity, smoking, and sleep) and four health factors (blood lipid, blood sugar, blood pressure, and body mass index). This study tried to evaluate the association between LE8 and cataract using data from National Health and Nutrition Examination Survey (NHANES) 2005–2008, a continuous research program which aims to monitor and evaluate the health and nutrition status of the US population. A cross-sectional study of 2720 non-cataract participants and 602 cataract participants. All participants were assigned to the poor, intermediate, and ideal CVH status groups based on LE8 score. Weighted multiple logistic regression was used to investigate the correlation between the LE8 score and cataract, as well as the correlation between each of the eight subitems and cataract, with potential confounding variables being adjusted. Then, restricted cubic spline analysis was used to further explore whether there was a nonlinear relationship between LE8 score and cataract. The proportion of cataract participants was 14.1%, 18.2%, and 20.6% in the ideal, intermediate, and poor CVH groups, respectively (P < 0.05). LE8 score was inversely associated with cataract risk, with each 10-point increase in LE8 score associated with a 14% reduction in cataract risk [odds ratio (OR) = 0.86, 95% confidence interval (CI): 0.79–0.93, P < 0.01]. Among all the LE8 subitems, physical activity, sleep, and blood glucose were significantly associated with cataract risk (all P < 0.05). Better CVH, defined by a higher LE8 score, is associated with a lower cataract risk. Efforts to improve LE8 score (especially when it comes to physical activity, sleep, and blood glucose) may serve as a novel strategy to help reduce the risk of cataract.

## Introduction

Cataract is a degenerative disease of the lens that is related to multiple factors such as age, environment, and metabolism^1–3^. In recent years, under the influence of the aging global population, the prevalence of cataracts has increased significantly^4^. In 2000, there were 20.5 million cataract patients in the United States, and this number is projected to increase to 38.7 million by 2030^5,6^. The harm caused by cataracts is mainly visual impairment, which can even lead to blindness in severe cases. According to the Global Burden of Disease Study, cataract is the leading cause of blindness in people aged over 50 years and the second leading global cause of moderate and severe vision impairment (MSVI) in the general population^7^. At present, surgery is still the only effective and recognized treatment option for cataracts. Modern cataract surgery, which includes removing the affected lens and implanting an artificial lens, has been shown to improve the patient's visual acuity and quality of life in a safe and quick manner. However, cataract surgery is not without surgical complications and has posed a considerable economic burden on healthcare systems^8^. In the United States, direct ophthalmic medical costs for cataract surgery in both eyes were $5052 per person^9^.

In 2010, the American Heart Association (AHA) introduced the concept of Life’s Simple 7 (LS7) as a tool for assessing cardiovascular health (CVH)^10^. LS7 comprises four modifiable behavioral factors (smoking, diet, physical activity [PA], and body mass index [BMI]) and three modifiable biological factors (blood pressure, total cholesterol, and fasting blood glucose)^10^. An elevated LS7 score indicates a favorable cardiovascular well-being status. Subsequent investigations indicated that people with higher LS7 scores not only had a lower risk of cardiovascular disease (CVD) but also a reduced risk of several non-CVDs (such as depression and cognitive dysfunction)^11–13^. In the field of ophthalmology, a previous study has shown that ideal CVH, as indicated by a higher LS7 score, is associated with reduced risks of some ocular diseases, particularly diabetic retinopathy^14^. This discovery implies that actively embracing healthy habits aligned with the LS7 framework in life is anticipated to lower the likelihood of developing eye conditions like diabetic retinopathy^14^. This finding suggests that actively cultivating healthy habits in accordance with the LS7 framework in life, is expected to reduce the probability of developing eye diseases such as diabetic retinopathy.

With the continuous accumulation and updating of empirical evidence, the AHA revised and reinforced the “LS7” framework in June 2022. This revision primarily incorporated sleep health into the framework, giving rise to the concept of “Life’s Essential 8 (LE8)”^15^. Each LE8 subitems has its own scoring criteria, and the final LE8 score is the average score of each subitem, ranging from 0 to 100. The higher the LE8 score, the better the CVH. Similar to LE7, higher LE8 scores are associated with a protective effect against stroke and CVD^16,17^. It has long been thought that certain CVD risk factors may also be associated with cataract risk, such as diabetes and obesity, both of which belong to the LE8 healthy factors. Meanwhile, sleep and PA, among the LE8 healthy behaviors, have received increasing attention in recent years as possible factors affecting cataract risk^18,19^. Thus, using LE8 factors as a metric to examine the relationship between these health variables and cataracts may provide valuable insights for cataract prevention^20,21^. However, to date, there is no published study concerning the correlation of the LE8 with cataracts. Given that surgery remains the only recognized effective treatment option for cataracts, it remains important to explore the modifiable risk factors for cataracts, which may provide new intervention strategies to delay or prevent the onset of cataracts, and finally to reduce the burden of this prevailing disease.

In this study, we conducted an analysis of the correlation between LE8 and cataracts employing data from the US National Health and Nutrition Examination Survey (NHANES) between 2005 and 2008, hopefully to provide healthy lifestyle guidance for cataract patients and high-risk groups.

## Materials and methods

### Research design and population

**Figure 1:**
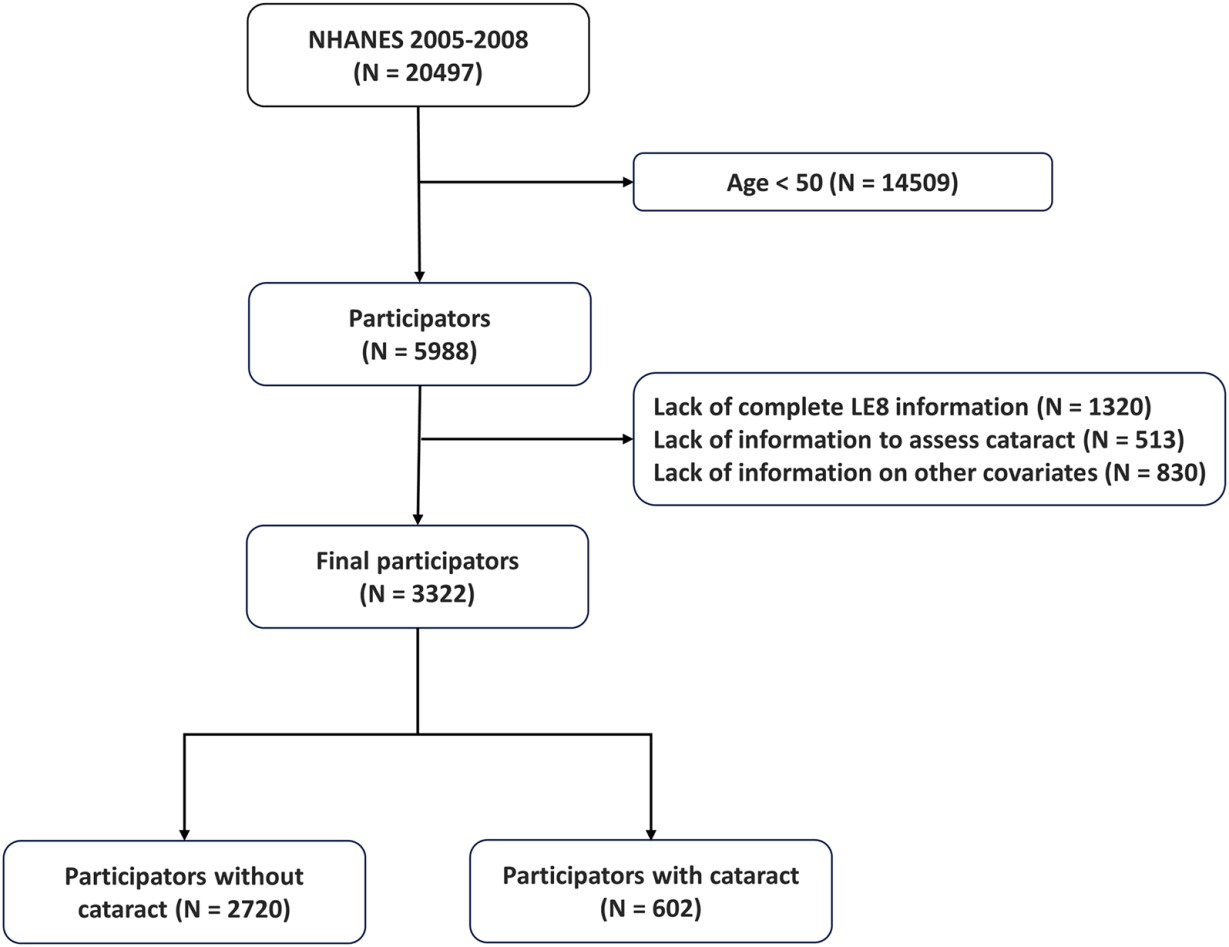
The flowchart of patient inclusion and exclusion.

NHANES is a government-sponsored continuous research program administered by the National Center for Health Statistics (NCHS), which aims to monitor and evaluate the health and nutrition status of the US population. The study protocol was approved by the Research Ethics Review Board of NCHS, and all methods were performed in accordance with the relevant guidelines and regulations. This survey adopts the method of multi-stage random sampling, which selects the residents of representative states in the US, and conducts personal interviews, physical measurements, laboratory examinations, and nutrition surveys, thus providing a large amount of data for the formulation of nutrition and health policies. In order to clarify the relationship between the LE8 and cataracts, the data of the NHANES database from 2005 to 2008 were selected in this study, with the specific exclusion criteria being as follows: (1) Age < 49 (n = 14,509); (2) lack of complete LE8 information (n = 1320); (3) lack of in-formation on cataract assessment (n = 513); (4) lack of information on other covariates, including gender, race, education level, household income level, and alcohol consumption (n = 830). Finally, 3322 participants with complete information comprised the study population, which included 2720 non-cataract participants and 602 cataract patients. Figure 1 shows the specific process for the inclusion of the study population.

### Definition of cataract

This study referred to other studies using self-reported history of cataract surgery as the diagnostic criteria^22,23^. Considering that the US has a coverage and low threshold for cataract surgery, self-reported cataract surgery could be used as a surrogate for clinically significant cataracts^22^. When participants completed a questionnaire that asked “Have you previously undergone cataract surgery?”. A positive response to this question was considered an indication of the presence of cataracts.

### Definition of LE8

The LE8 considers the impact of both health behaviors and health factors on CVH^15^. Healthy behaviors include diet, PA, smoking (nicotine exposure), and sleep health, while healthy factors include blood lipid (non-high-density lipoprotein cholesterol [non-HDL cholesterol]), blood sugar, blood pressure, and BMI. The specific distribution scores of each subitem are shown in Supplementary Table 1 and Table 2^15^. The average of the scores of the 8 subitems is the final LE8 score, which ranges between 0 and 100. As per the AHA, a score of 80–100 indicates high CVH, while a score of 50–79 suggests moderate CVH, and a score of 0–49 indicates low CVH^15^. In addition, we also divided each of the eight subitems into three levels: Ideal, Intermediate, and Poor. Classification criteria are listed in Table 1.

**Table 1:** The classification criteria of the 8 subitems in LE8.

	Subitems	Classification	Criteria
Healthy behaviors	Diet	Poor	1th–49th% quantile
		Intermediate	50th–94th% quantile
		Ideal	95th–100th% quantile
	PA	Poor	No activity
		Intermediate	1–149 min of moderate/vigorous PA per wk
		Ideal	≥ 150 min of moderate/vigorous PA per wk
	Smoking (nicotine exposure)	Poor	Current smoker
		Intermediate	Former smoker
		Ideal	Never smoker
	Sleep health (average sleep duration)	Poor	< 6 h/d or ≥ 10 h/d
		Intermediate	70–90 6–7 h/d or 9–10 h/d
		Ideal	7–9 h/d
Healthy factors	Blood lipid (non-HDL cholesterol)	Poor	≥ 160 mg/dL
		Intermediate	130–159 mg/dL
		Ideal	< 130 mg/dL
	Blood glucose	Poor	HbA1c ≥ 6.5%
		Intermediate	FBG 100–125 mg/dL or HbA1c: 5.7–6.4%
		Ideal	FBG < 100 mg/dL or HbA1c < 5.7% with no diabetes history
	Blood pressure	Poor	SBP ≥ 140 mmHg or DBP ≥ 90 mmHg
		Intermediate	120–129/ < 80 mmHg or SBP 130–139 mmHg or DBP 80–89 mmHg
		Ideal	SBP < 120 mmHg and DBP < 80 mmHg
	BMI	Poor	≥ 30 kg/m^2^
		Intermediate	25–29.9 kg/m^2^
		Ideal	< 25 kg/m^2^

LE8, Life’s Essential 8; HEI-2015, Healthy Eating Index-2015; PA, physical activity; non-HDL cholesterol, non-high density lipoprotein cholesterol; FBG, fasting blood glucose; HbA1c, glycated hemoglobin; SBP, systolic blood pressure; DBP, diastolic blood pressure; BMI, body mass index.

### Definition of covariates

According to relevant studies^14,24^, we adjusted for some risk factors that might have affected the outcome as covariates. These factors mainly included: (1) age; (2) gender (male and female); (3) race (white, black, Mexican American, and other races); (4) education level (below high school, high school, and more than high school); (5) household income level, which was used household in-come-to-poverty line ratio (< 1.3, 1.3–3.5, and > 3.5); (6) alcohol consumption (never, former, moderate, mild, and heavy). Since some factors such as blood pressure, BMI, blood lipid, and diabetes were included in the estimates of the LE8 score, we did not make additional adjustments for these factors.

### Statistical analysis

To ensure that the data are representative of the whole US population, our analysis was weighted according to the weights recommended by the NCHS^25^. In the initial phase, the data were comprehensively summarized and expounded upon based on the presence or absence of cataract. For categorical variables (expressed as percentages), the comparison between the two groups was conducted utilizing Chi-square tests, whereas continuous variables (reported as mean ± standard deviation) were subjected to one-way analysis of variance for between-group comparisons. We employed weighted multiple logistic regression to investigate the correlation between the overall LE8 score and cataract, as well as the link between each of the eight subitems and cataract. Subsequently, we adjusted for several potential confounding variables (age, gender, race, education level, household income level, and alcohol consumption) to enhance the robustness of the findings. In addition, restricted cubic spline (RCS) analysis was used to further explore whether there was a nonlinear relationship between LE8 score and cataract.

All analyses were based on survey and rms packages in R software (version 4.2.3), where P < 0.05 was considered statistically significant.

### Ethics approval and consent to participate

The NCHS Institutional Review Board has approved NHANES's investigative, and all participants have provided written informed consent.

## Results

### Population baseline characteristics

The survey-weighted characteristics of the study population are summarized in Table 2 according to the CVH status of the participants. Overall, the mean age of all participants was 65.37 years, among whom 49.8% were females. A total of 602 cataract patients were included. The LE8 score of all participants was 62.86 ± 13.64, whereas the poor, intermediate, and ideal CVH groups had scores of 41.46 ± 6.22, 64.32 ± 8.15, and 85.06 ± 4.15, respectively (P < 0.01). The proportion of cataract participants was 14.1%, 18.2%, and 20.6% in the ideal, intermediate, and poor CVH group, respectively (P < 0.05). No significant difference in age was found among the three groups (P > 0.05). There were significant differences among the three groups in terms of household income level, gender, race, educational level, and alcohol consumption (all P < 0.05).

**Table 2:** Population baseline characteristics according to cardiovascular health status.

Variables	Total (n = 3322)	Poor (n = 540)	Intermediate (n = 2421)	Ideal (n = 361)	P value
LE8 Score [mean (SD)]*	62.86 (13.64)	41.46 (6.22)	64.32 (8.15)	85.06 (4.15)	< 0.01
Cataract (%)					
Yes	602 (18.1)	111 (20.6)	440 (18.2)	51 (14.1)	< 0.05
No	2720 (81.9)	429 (79.4)	1981 (81.8)	310 (85.9)	
Age [mean (SD)]*	65.37 (9.81)	65.16 (9.15)	65.52 (9.96)	64.74 (9.72)	> 0.05
Age group (%)					> 0.05
< 65	1667 (60.7)	282 (58.7)	1201 (60.6)	184 (63.7)	
65–76	1254 (30.9)	207 (32.9)	909 (30.7)	138 (30.0)	
> = 80	401 (8.4)	51 (8.4)	311 (8.7)	39 (6.4)	
Poverty [mean (SD)]*	2.78 (1.59)	2.15 (1.43)	2.83 (1.58)	3.41 (1.57)	< 0.01
Household income level (%)					< 0.01
Poor (PIR < 1.3)	793 (23.9)	201 (37.2)	543 (22.4)	49 (13.6)	
Moderate (PIR 1.3–3.5)	1348 (40.6)	237 (43.9)	988 (40.8)	123 (34.1)	
High (PIR > 3.5)	1181 (35.6)	102 (18.9)	890 (36.8)	189 (52.4)	
Gender (%)					< 0.05
Male	1668 (50.2)	243 (45.0)	1253 (51.8)	172 (47.6)	
Female	1654 (49.8)	297 (55.0)	1168 (48.2)	189 (52.4)	
Race (%)					< 0.01
White	1971 (59.3)	263 (48.7)	1449 (59.9)	259 (71.7)	
Black	622 (18.7)	169 (31.3)	422 (17.4)	31 (8.6)	
Mexican American	436 (13.1)	66 (12.2)	333 (13.8)	37 (10.2)	
Other	293 (8.8)	42 (7.8)	217 (9.0)	34 (9.4)	
Educational level (%)					< 0.01
Below high school	473 (14.2)	116 (21.5)	327 (13.5)	30 (8.3)	
High School	1352 (40.7)	259 (48.0)	996 (41.1)	97 (26.9)	
More than high school	1497 (45.1)	165 (30.6)	1098 (45.4)	234 (64.8)	
Dietary (%)					< 0.01
Poor	1374 (41.4)	385 (71.3)	970 (40.1)	19 (5.3)	
Intermediate	1690 (50.9)	152 (28.1)	1282 (53.0)	256 (70.9)	
Ideal	258 (7.8)	3 (0.6)	169 (7.0)	86 (23.8)	
Physical activity (%)					< 0.01
Poor	1089 (32.8)	396 (73.3)	687 (28.4)	6 (1.7)	
Intermediate	821 (24.7)	87 (16.1)	652 (26.9)	82 (22.7)	
Ideal	1412 (42.5)	57 (10.6)	1082 (44.7)	273 (75.6)	
Smoking (%)					< 0.01
Poor	548 (16.5)	202 (37.4)	341 (14.1)	5 (1.4)	
Intermediate	1244 (37.4)	175 (32.4)	959 (39.6)	110 (30.5)	
Ideal	1530 (46.1)	163 (30.2)	1121 (46.3)	246 (68.1)	
Sleep duration (%)					< 0.01
Poor	556 (16.7)	194 (35.9)	355 (14.7)	7 (1.9)	
Intermediate	953 (28.7)	175 (32.4)	710 (29.3)	68 (18.8)	
Ideal	1813 (54.6)	171 (31.7)	1356 (56.0)	286 (79.2)	
Body mass index (%)					< 0.01
Poor	548 (16.5)	202 (37.4)	341 (14.1)	5 (1.4)	
Intermediate	1244 (37.4)	175 (32.4)	959 (39.6)	110 (30.5)	
Ideal	1530 (46.1)	163 (30.2)	1121 (46.3)	246 (68.1)	
Non-HDL cholesterol (%)					< 0.01
Poor	1427 (43.0)	325 (60.2)	1038 (42.9)	64 (17.7)	
Intermediate	1364 (41.1)	186 (34.4)	1006 (41.6)	172 (47.6)	
Ideal	531 (16.0)	29 (5.4)	377 (15.6)	125 (34.6)	
Blood glucose (%)					< 0.01
Poor	630 (19.0)	231 (42.8)	392 (16.2)	7 (1.9)	
Intermediate	892 (26.9)	190 (35.2)	672 (27.8)	30 (8.3)	
Ideal	1800 (54.2)	119 (22.0)	1357 (56.1)	324 (89.8)	
Blood pressure (%)					< 0.01
Poor	1261 (38.0)	330 (61.1)	891 (36.8)	40 (11.1)	
Intermediate	1527 (46.0)	196 (36.3)	1160 (47.9)	171 (47.4)	
Ideal	534 (16.1)	14 (2.6)	370 (15.3)	150 (41.6)	
Alcohol consumption (%)					< 0.01
Never	506 (15.2)	77 (14.3)	367 (15.2)	62 (17.2)	
Former	960 (28.9)	217 (40.2)	678 (28.0)	65 (18.0)	
Moderate	399 (12.0)	66 (12.2)	281 (11.6)	52 (14.4)	
Mild	1182 (35.6)	129 (23.9)	885 (36.6)	168 (46.5)	
Heavy	275 (8.3)	51 (9.4)	210 (8.7)	14 (3.9)	

LE8, Life's Essential 8; SD, standard deviation; PIR, Family income to poverty ratio; non-HDL cholesterol, non-high density lipoprotein cholesterol.

*Between-group comparisons were conducted using one-way analysis of variance. Others: between-group comparisons were conducted using Chi-square tests.

### Relationship between LE8 score and cataract

In Table 3, after adjusting for covariates (age, gender, and race; Model 1), the presented odds ratio (OR) showed that every 10-point increase in the LE8 score was associated with a 15% decrease in the risk of having cataract (OR = 0.85, 95% CI: 0.79–0.92, P < 0.01). Even after continuing to adjust for education level, household income level, and alcohol consumption (Model 2), the result remained stable (OR = 0.86, 95% CI: 0.79–0.93, P < 0.01). When classified according to the CVH status recommended by the AHA, in Model 1, individuals with moderate to high CVH levels exhibited a reduced risk of cataract in comparison to those with low CVH levels (moderate CVH: OR = 0.71, 95% CI: 0.54–0.93, P < 0.001; High CVH: OR = 0.52, 95% CI: 0.34–0.79, P < 0.01). However, in Model 2, only highly cardiovascular-healthy individuals had a lower risk of cataract (OR = 0.56, 95% CI: 0.36–0.85, P < 0.01).

**Table 3:** Relationship between LE8 score and cataract.

	Model 1		Model 2	
	OR (95% CI)	P value	OR (95% CI)	P value
LE8 score (Per 10 points increase)	0.85 (0.79, 0.92)	< 0.01	0.86 (0.79, 0.93)	< 0.01
LE8 score classification				
Poor CVH	ref	ref	ref	ref
Moderate CVH	0.71 (0.54, 0.93)	< 0.05	0.74 (0.56, 0.98)	> 0.05
Ideal CVH	0.52 (0.34, 0.79)	< 0.01	0.56 (0.36, 0.85)	< 0.01
P for trend		< 0.01		< 0.01

Model 1: Adjust for age, gender, and race.

Model 2: Adjust for age, gender, race, education level, household income level, and alcohol consumption.

LE8, Life's Essential 8; OR, odds ratio; CI, confidence interval; CVH, cardiovascular health.

Analyses were conducted using weighted multivariable logistics regression.

We further performed subgroup analysis and RCS analysis to determine whether the protective effect of LE8 score on cataract differs among different populations. Subgroup analysis showed that LE8 scores were more protective for individuals aged ≥ 65 years than those aged < 65 years, and were more protective for Caucasians and Mexican Americans than for African Americans and other races (Supplementary Table 3). The RCS showed a linear negative relationship between the LE8 score and cataract risk, i.e., the higher the LE8 score, the lower the risk of cataract, and this trend remained consistent across genders and age groups (Supplementary Fig. 1).

### Relationship between health behaviors/factors and cataract

We further analyzed the relationship between each LE8 subitem and cataract (Table 4).

**Table 4:** Relationship between health behavior and factor score and cataract.

		Model1		Model2	
		OR (95% CI)	P	OR (95% CI)	P value
Healthy behaviors	Diet				
	Poor	ref	ref	ref	ref
	Intermediate	0.94 (0.76, 1.17)	> 0.05	0.96 (0.77, 1.20)	> 0.05
	Ideal	0.67 (0.45, 0.97)	< 0.05	0.68 (0.46, 1.00)	> 0.05
	P for trend		> 0.05		> 0.05
	Physical activity				
	Poor	ref	ref	ref	ref
	Intermediate	0.62 (0.47, 0.81)	< 0.01	0.65 (0.49, 0.85)	< 0.01
	Ideal	0.68 (0.54, 0.86)	< 0.01	0.71 (0.56, 0.90)	< 0.01
	P for trend		< 0.01		< 0.01
	Nicotine exposure				
	Poor	ref	ref	ref	ref
	Intermediate	1.20 (0.85, 1.73)	> 0.05	1.25 (0.87, 1.81)	> 0.05
	Ideal	1.09 (0.77, 1.57)	> 0.05	1.10 (0.76, 1.61)	> 0.05
	P for trend		> 0.05		> 0.05
	Sleep duration				
	Poor	ref	ref	ref	ref
	Intermediate	0.77 (0.57, 1.04)	> 0.05	0.79 (0.58, 1.08)	> 0.05
	Ideal	0.70 (0.53, 0.92)	< 0.05	0.72 (0.55, 0.96)	< 0.05
	P for trend		< 0.05		< 0.05
Healthy factors	BMI				
	Poor	ref	ref	ref	ref
	Intermediate	1.20 (0.85, 1.73)	> 0.05	1.25 (0.87, 1.81)	> 0.05
	Ideal	1.09 (0.77, 1.57)	> 0.05	1.10 (0.76, 1.61)	> 0.05
	P for trend		> 0.05		> 0.05
	Blood pressure				
	Poor	ref	ref	ref	ref
	Intermediate	0.85 (0.69, 1.06)	> 0.05	0.86 (0.69, 1.07)	> 0.05
	Ideal	0.96 (0.67, 1.37)	> 0.05	0.98 (0.68, 1.39)	> 0.05
	P for trend		> 0.05		> 0.05
	Blood glucose				
	Poor	ref	ref	ref	ref
	Intermediate	0.42 (0.32, 0.57)	< 0.01	0.43 (0.32, 0.58)	< 0.01
	Ideal	0.45 (0.35, 0.59)	< 0.01	0.46 (0.35, 0.60)	< 0.01
	P for trend		< 0.01		< 0.01
	Non-HDL cholesterol				
	Poor	ref	ref	ref	ref
	Intermediate	1.06 (0.85, 1.32)	> 0.05	1.06 (0.85, 1.32)	> 0.05
	Ideal	1.18 (0.87, 1.59)	> 0.05	1.16 (0.85, 1.57)	> 0.05
	P for trend		> 0.05		> 0.05

Model 1: Adjust for age, gender, and race.

Model 2: Adjust for age, gender, race, education level, poverty status, and alcohol consumption.

LE8,Life's Essential 8; OR, odds ratio; CI, confidence interval; BMI, body mass index; non-HDL cholesterol, non-high density lipoprotein cholesterol.

Analyses were conducted using weighted multivariable logistics regression.

Among the four healthy behaviors, ideal sleep was negatively correlated with the risk of cataract [Model 1: OR = 0.70, 95% CI: 0.53–0.92, P < 0.05; Model 2: OR = 0.72, 95% CI: 0.55–0.96, P < 0.05]. Both intermediate PA [Model 1: OR = 0.62, 95% CI: 0.47–0.81, P < 0.01; Model 2: OR = 0.65, 95% CI: 0.49–0.85, P < 0.01] and ideal PA [Model 1: OR = 0.68, 95% CI: 0.54–0.86, P < 0.01; Model 2: OR = 0.71, 95% CI: 0.56–0.90, P < 0.01] significantly reduced the risk of cataract. Notably, ideal diet had a protective effect against cataracts in Model 1, whereas this protective effect was absent in model 2 [Model 1: OR = 0.67, 95% CI: 0.45–0.97, P < 0.05; Model 2: OR = 0.68, 95% CI: 0.46–1.00, P > 0.05]. Besides, we observed no significant effect of smoking on cataracts.

As for the four healthy factors, we observed that only intermediate blood glucose status [Model 1: OR = 0.42, 95% CI: 0.32–0.57, P < 0.01; Model 2: OR = 0.43, 95% CI: 0.32–0.58, P < 0.01] and ideal blood glucose status [Model 1: OR = 0.45, 95% CI: 0.35–0.59, P < 0.01; Model 2: OR = 0.46, 95% CI: 0.35–0.60, P < 0.01] were negatively associated with cataract risk.

## Discussion

This study, to our knowledge, is the first to analyze the correlation between LE8 score (as well as each LE8 subitem) and the risk of cataracts. We found that the LE8 score was inversely associated with cataract risk. Among all the LE8 subitems, sleep duration, physical activity, and blood glucose were significantly associated with cataract risk. Our findings provide new insight into the strategy to reduce cataract and its accompanying disease burden.

Currently, a comprehensive assessment of the relationship between ideal CVH indicators and cataract risk is lacking, although some previous studies have observed associations between some LE8 subitems (such as blood glucose and diet) and cataracts^26,27^. Therefore, we first analyzed the correlation between LE8, an overall measure of CVH, and cataracts. We found that the better the CVH status, the lower the proportion of cataract patients, and every 10-point increase in the LE8 score was associated with a 14% reduction in cataract risk, both of which suggest that better CVH is associated with lower cataract risk. To better describe whether there is a non-linear relationship between LE8 scores and cataracts, we then performed an RCS analysis. In the RCS analysis, the LE8 score was negatively correlated with cataract risk with a cut-off LE8 score of 63, that is, people with this score have the same risk of cataracts as the general population, whereas those with a score higher than 63 have a relatively lower risk. These findings suggest that efforts to improve CVH (reflected in a higher LE8 score) can serve as a novel strategy to help reduce the risk of cataracts.

Considering that all the eight subitems of LE8 score could be controlled by lifestyle modification or medical treatment, we conducted further analyses to determine which subitems have the most significant impact on cataract risk. We found that intermediate-to-ideal-intensity PA, ideal sleep, and intermediate-to-ideal blood glucose status were associated with a reduced risk of cataracts.

The direct cause of cataract development is unknown, but oxidative damage is thought to play an important role in its pathogenesis^28^. Reactive oxygen species (ROS) can induce damage to the lens cell via different ways, thus causing opacification of the lens, namely cataracts^29^. PA may reduce oxidative stress levels by increasing endogenous antioxidant defenses^30^. A large meta-analysis involving 6 cohort studies also found that increased PA is negatively related to cataract risk in a dose-responsive manner^18^. This relationship is plausible given the beneficial effects of physical activity on oxidative stress.

Previous studies indicate that individuals with inadequate sleep duration are more prone to cataracts, potentially due to reduced resilience to oxidative stress, prolonged exposure to ultraviolet (UV) rays, and an increased likelihood of diabetes or hypertension, all of which are established risk factors for cataracts^19,31–33^. However, there are currently limited studies evaluating the relationship between sleep and cataracts, so more research is needed.

Of note, it has been noticed that there is an interaction between screen time and sleep duration, sleep quality, as well as PA^34–36^. Specifically, prolonged screen time correlates with shortened sleep duration, diminished sleep quality, and reduced PA. Conversely, increased PA and sufficient sleep would naturally allocate individuals less time for screen usage. From this perspective, excessive screen time might indirectly elevate the risk of cataracts by impacting both sleep patterns and PA levels. Furthermore, exposure to blue light from LED (light-emitting diode) screens may have a direct promoting effect on cataracts^37,38^. In this era of escalating screen use, the relationship between screen time and cataracts merits in-depth exploration through large-scale cohort studies.High blood sugar level (including diabetes) has long been recognized as an important risk factor for cataract occurrence and progression^26^. The pathogenesis of diabetic cataracts is complex and involves many factors. Long-term experimental studies and clinical observations have put forward a variety of different hypotheses on its pathogenesis. At present, the mainstream theories mainly include three theories. First is the polyol pathway. Aldose reductase is a key enzyme in diabetic cataracts, as it plays an important role in the polyol pathway^40^. When glucose in aqueous humor reaches saturation, aldose reductase is activated, converting glucose into sorbitol and fructose. Sorbitol can increase the osmotic pressure in the lens after accumulation, causing excessive water to enter the lens, resulting in cataracts^41,42^. The second is oxidative stress. Lens epithelial cells maintain the stability and transparency of the lens. ROS, however, induces apoptosis of lens epithelial cells, causing diabetic cataract^43^. The third is non-enzymatic glycation. In the physiological state, only a small amount of glycosylation products (AGE) exists in the lens, but in hyperglycemic environment, AGE increases and accumulates in large quantities, which promotes cataract formation through various pathways^44,45^.

Notably, in Model 1, we found that ideal dietary status was significantly associated with a lower risk of cataract. In LE8, “diet” refers specifically to the HEI-2015 (Healthy Eating Index-2015) diet, a healthy eating pattern proposed by Dietary Guidelines for Americans. A large cohort study in the UK showed that there was a strong relationship between cataract risk and diet patterns, with a gradual reduction in cataract risk among high meat eaters to low meat eaters, fish eaters (participants who ate fish instead of meat), and vegetarians^27^. Zhou et al.^46^ also found that adherence to the healthy eating patterns advocated by HEI-2015 was associated with a reduced risk of age-related cataracts. However, in Model 2, diet was not statistically associated with cataract risk. Considering that the relationship between diet and health is extremely complex, its correlation with cataract needs to be further verified.There are two most notable strengths in this study. One is the novelty (the first to provide a comprehensive assessment of the relationship between LE8 and cataract). The other is that we have identified three modifiable behaviors and factors (PA, sleep, and blood glucose), which have guiding effects on reducing the risk of cataracts for the public.

This study has some limitations. First, LE8 is a comprehensive indicator of cardiovascular health, and its effect on cataracts may be caused by the combination of the eight subitems it contains. Therefore, although we found that the ideal state of the three subitems had a statistically significant protective effect on cataract, this does not necessarily mean that the other subitems do not have an effect on cataract. Second, in this study, diagnosis of cataract is based on self-reported cataract surgeries. While this approach has been reported as a good indicator of the presence of clinically significant cataract, it may underestimate the burden of cataract. Third, due to the limitations of the NHANES database, we did not exclude individuals with other eye diseases, and we call for more well-designed studies to address this issue in the future.

## Conclusions

Better CVH is associated with a lower cataract risk. Adhering to the healthy lifestyle recommended by LE8 (especially when it comes to PA, sleep, and blood glucose) may reduce the occurrence of cataracts. Our findings suggest that interventions to prevent cardiovascular diseases hold promise for reducing the disease burden of cataract.

### Supplementary Information

Supplementary Figure 1.

Supplementary Tables.

## Data Availability

The datasets for this study can be found in the National Health and Nutrition Examination Surveys database (https://www.cdc.gov/nchs/nhanes/index.htm).
