# Supplementary material for: Association between Life’s Essential 8 and cataract among US adults
Source: Sci Rep. 2024 Jun 7;14:13101. doi: 10.1038/s41598-024-63973-1 (PMC11161494; doi:10.1038/s41598-024-63973-1)
Supplement: Supplementary file 1 — Supplementary Figure 1. [file 41598_2024_63973_MOESM1_ESM.pdf]

Supplementary Figure 1. RCS analysis between LE8 score and risk of cataract.

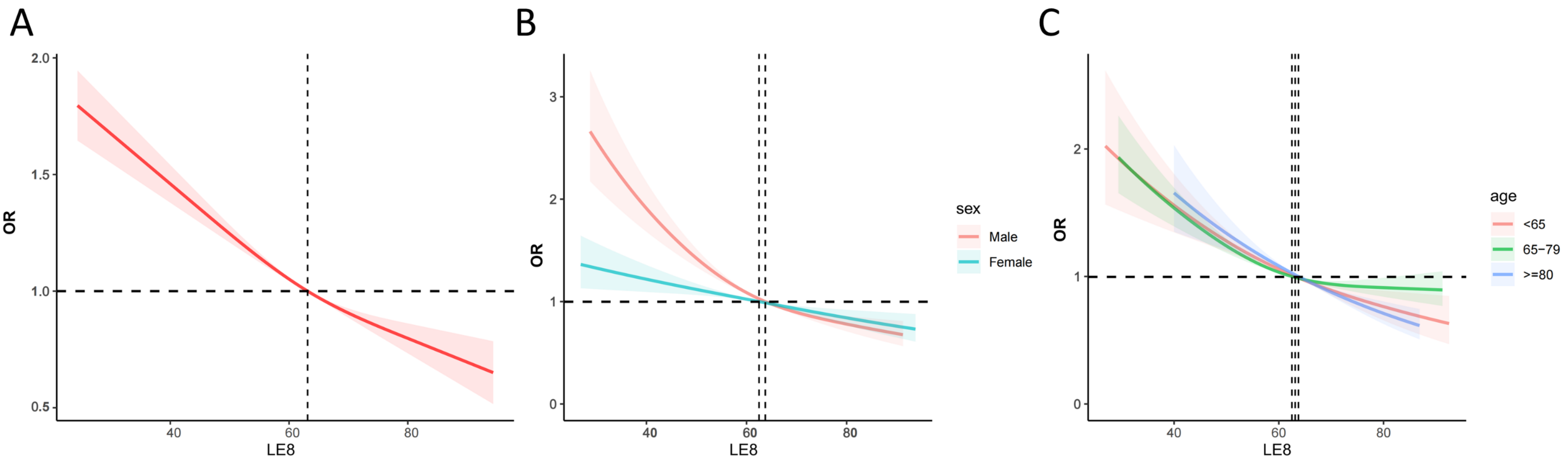

A. All-individual RCS analysis. The cut-off value is 63.

B. Sex-stratified RCS analysis. The cut-off value is 62 for females and 64 for males, respectively.

C. Age-stratified RCS analysis. The cut-off value is 63 for individuals aged < 65, 62 for individuals aged 65-79, and 64 for individuals aged≥80, respectively.

RCS=Restricted cubic spline; LE8=Life's Essential 8; OR=odds ratio.

All P for non-linear > 0.05.
