# Supplementary material for: Association between Life’s Essential 8 and cataract among US adults
Source: Sci Rep. 2024 Jun 7;14:13101. doi: 10.1038/s41598-024-63973-1 (PMC11161494; doi:10.1038/s41598-024-63973-1)
Supplement: Supplementary file 2 — Supplementary Tables. [file 41598_2024_63973_MOESM2_ESM.pdf]

Supplementary Table 1. Definition and scoring approach for quantifying cardiovascular health, as per the American Heart Association's Life's Essential 8 score.

Domain	CVH Metric	Measurement	Quantification and Scoring of CVH Metric-Adults (≥20 Years)
**Health Behaviors**	**Diet**	**Healthy Eating Index-2015 (HEI-2015) diet score percentile**	**Metric: Quantiles of HEI-2015-style diet adherence**<br><br>Scoring (Population):<br>Points Quantile<br>100 ≥95th percentile (top/ideal diet)<br>80 75th – 94th percentile<br>50 50th – 74th percentile<br>25 25th – 49th percentile<br>0 1st – 24th percentile (bottom/least ideal quartile)
	**Physical activity**	**Self-reported minutes of moderate or vigorous physical activity per week**	**Metric: Minutes of moderate (or greater) intensity activity per week**<br><br>Scoring:<br>Points Minutes<br>100 ≥150<br>90 120 – 149<br>80 90 – 119<br>60 60 – 89<br>40 30 – 59<br>20 1 – 29<br>0 0
	**Nicotine exposure**	**Self-reported use of cigarettes or inhaled nicotine-delivery system (NDS)**	**Metric: Combustible tobacco use and/or inhaled NDS use; or secondhand smoke exposure**<br><br>Scoring:<br>Points Status<br>100 Never smoker<br>75 Former smoker, quit ≥5 yrs<br>50 Former smoker, quit 1 - <5 yrs

			25 Former smoker, quit <1 year, or currently using inhaled NDS<br>0 Current smoker<br><br>Subtract 20 points (unless score is 0) for living with active indoor smoker in home
	**Sleep health**	**Self-reported average hours of sleep per night**	**Metric: Average hours of sleep per night**<br><br>Scoring:<br>Points Level<br>100 7 – <9<br>90 9 – <10<br>70 6 – <7<br>40 5 – <6 or ≥10<br>20 4 – <5<br>0 <4
**Health Factors**	**Body mass index**	**Body weight (kg) divided by height squared (m^2)**	**Metric: Body mass index (kg/m^2)**<br><br>Scoring: Points Level<br>100 <25<br>70 25.0 – 29.9<br>30 30.0 – 34.9<br>15 35.0 – 39.9<br>0 ≥40.0
	**Blood lipids**	**Plasma total and high-density lipoprotein-cholesterol (HDL-cholesterol) with calculation of non-HDL-cholesterol**	**Metric: Non-HDL-cholesterol (mg/dL)**<br><br>Scoring:<br>Points Level<br>100 <130<br>60 130 – 159<br>40 160 – 189<br>20 190 – 219<br>0 ≥220<br><br>If drug-treated level, subtract 20 points

	**Blood glucose**	**Fasting blood glucose or casual hemoglobin A1c**	**Metric: Fasting blood glucose (mg/dL) or Hemoglobin A1c (%)**<br><br>Scoring:<br>Points Level<br>100 No history of diabetes and FBG <100 (or HbA1c < 5.7)<br>60 No diabetes and FBG 100 – 125 (or HbA1c 5.7-6.4) (Pre-diabetes)<br>40 Diabetes with HbA1c <7.0<br>30 Diabetes with HbA1c 7.0 – 7.9<br>20 Diabetes with HbA1c 8.0 – 8.9<br>10 Diabetes with Hb A1c 9.0 – 9.9<br>0 Diabetes with HbA1c ≥10.0
	**Blood pressure**	**Appropriately measured systolic and diastolic blood pressure**	**Metric: Systolic and diastolic blood pressure (mm Hg)**<br><br>Scoring:<br>Points Level<br>100 <120/<80 (Optimal)<br>75 120-129/<80 (Elevated)<br>50 130-139 or 80-89 (Stage I HTN)<br>25 140-159 or 90-99<br>0 ≥160 or ≥100<br><br>Subtract 20 points if treated level

Reference

Lloyd-Jones DM, Allen NB, Anderson CAM, et al. Life's Essential 8: Updating and Enhancing the American Heart Association's Construct of Cardiovascular Health: A Presidential Advisory From the American Heart Association. Circulation. 2022;146(5):e18-e43.

Supplementary Table 2. Definition and scoring approach for Healthy Eating Index-2015 diet score.

Component	Maximum points	Standard for maximum score	Standard for minimum score of zero
**Adequacy**			
Total Fruits	5	≥0.8 cup equiv. per 1,000 kcal	No fruit
Whole Fruits	5	≥0.4 cup equiv. per 1,000 kcal	No whole fruit
Total Vegetables	5	≥1.1 cup equiv. per 1,000 kcal	No vegetables
Greens and Beans	5	≥0.2 cup equiv. per 1,000 kcal	No dark green vegetables or legumes
Whole Grains	10	≥1.5 oz equiv. per 1,000 kcal	No whole grains
Dairy	10	≥1.3 cup equiv. per 1,000 kcal	No dairy
Total Protein Foods	5	≥2.5 oz equiv. per 1,000 kcal	No protein foods
Seafood and Plant Proteins	5	≥0.8 oz equiv. per 1,000 kcal	No seafood or plant proteins
Fatty Acids	10	(PUFAs + MUFAs)/SFAs ≥2.5	(PUFAs + MUFAs)/SFAs ≤1.2
**Moderation**			
Refined Grains	10	≤1.8 oz equiv. per 1,000 kcal	≥4.3 oz equiv. per 1,000 kcal
Sodium	10	≤1.1 gram per 1,000 kcal	≥2.0 grams per 1,000 kcal
Added Sugars	10	≤6.5% of energy	≥26% of energy
Saturated Fats	10	≤8% of energy	≥16% of energy

PUFAs=polyunsaturated fatty acids; MUFAs=monounsaturated fatty acids; SFAs-saturated fatty acids.

Adequacy components represent the food groups, subgroups, and dietary elements that are encouraged. For these components, higher scores reflect higher intakes, because higher intakes are desirable. Moderation components represent the food groups and dietary elements for which there are recommended limits to consumption. For moderation components, higher scores reflect lower intakes, because lower intakes are more desirable.

Reference

1. Krebs-Smith SM, Pannucci TE, Subar AF, et al. Update of the Healthy Eating Index: HEI-2015. J Acad Nutr Diet. 2018;118(9):1591-1602.

2. Wang L, Wang S, Wang Y, et al. Association between dietary live microbe intake and Life's Essential 8 in US adults: a cross-sectional study of NHANES 2005-2018. Front Nutr. 2024;11:1340028.

Supplementary Table 3. Subgroup analysis of the effect of cardiovascular health status on cataract in different populations.

Subgroup variables	OR* (95% CI)	P	P for interaction
Gender			>0.05
Male	0.88 (0.78,0.99)	<0.05	
Female	0.88 (0.79,0.98)	<0.05	
Age			>0.05
<65	0.90 (0.72,1.13)	>0.05	
65-79	0.88 (0.78,1.00)	<0.05	
≥80	0.80 (0.65,0.98)	<0.05	
Race			>0.05
White	0.86 (0.78,0.96)	<0.01	
Black	0.87 (0.71,1.08)	>0.05	
Mexican American	0.75 (0.56,0.99)	<0.05	
Other	0.84 (0.64,1.09)	>0.05	

OR=odds ratio; CI=confidence interval.

* the value corresponding to every 10-point increase in Life's Essential 8 score.
